# Primary Wilms tumor of the urinary bladder

**DOI:** 10.4322/acr.2021.390

**Published:** 2022-07-18

**Authors:** Mayur Parkhi, Srinivasan Peyam, Nitin James Peters, Kushaljit Singh Sodhi, Amita Trehan, Amanjit Bal

**Affiliations:** 1 Post Graduate Institute and Medical Education and Research, Department of Histopathology, Chandigarh, India; 2 Post Graduate Institute and Medical Education and Research, Department of Pediatric, Chandigarh, India; 3 Post Graduate Institute and Medical Education and Research, Department of Pediatric Surgery, Chandigarh, India; 4 Post Graduate Institute and Medical Education and Research, Department of Radiodiagnosis and Imaging, Chandigarh, India

**Keywords:** Wilms Tumor, Urinary Bladder, Antineoplastic Agents, Cystectomy

## Abstract

Wilms tumor (WT) can occur at various extrarenal sites; however, the urinary bladder as the primary site is occasional. A 4-year-old-female child presented with difficulty in micturition for the past month. The contrast-enhanced magnetic resonance imaging with magnetic resonance (MR) urography revealed a polypoidal, heterogeneous mass in the urinary bladder with no abnormality in the kidneys. Cystoscopy-guided biopsy was reported as an extrarenal Wilms tumor (ERWT) with triphasic components. Post-chemotherapy, a computed tomography scan revealed a residual tumor for which she underwent partial cystectomy. The diagnosis of ERWT was confirmed. She received adjuvant chemotherapy and remained well at the 9^th^ month post completion of chemotherapy. The primary bladder WT must be considered in the differential of a small blue round cell tumor at an extrarenal site in the pediatric age group. The diagnosis is especially challenging in small biopsy material, although it has immense significance in management and prognosis.

## INTRODUCTION

Wilms tumor (WT) or nephroblastoma, derived from nephrogenic blastemal cells, is the most common pediatric solid renal tumor occurring before 6 years in 90% of the cases.^1^ The classic location for WT is the kidney; however, it can present as primary at extrarenal sites including retroperitoneum, sacrococcygeal region, testis, uterus, inguinal canal, and mediastinum.^2^ Among these extrarenal locations, the urinary bladder as the primary site is extremely uncommon. To be more precise, we did a thorough literature search in the advanced section of the PubMed database using the combination of key terms: 'Wilms tumor' OR 'Nephroblastoma' AND 'urinary bladder' AND 'extrarenal'. The reference column of all the retrieved articles was also looked at. To date, only three cases have been registered in the English literature. In 2010, Zhang et al.^3^ reported the first case of WT occurring at two distinct primary sites (left kidney and the bladder) and presumed that they developed independently. The other two cases reported the urinary bladder as the primary site while the kidneys were uninvolved.^4^^,^ 5 Herein, we present another rare case of primary extrarenal WT in a 4-year-old-female child with urinary bladder as the primary site for WT and no evidence of renal involvement.

## CASE REPORT

**Figure 1:**
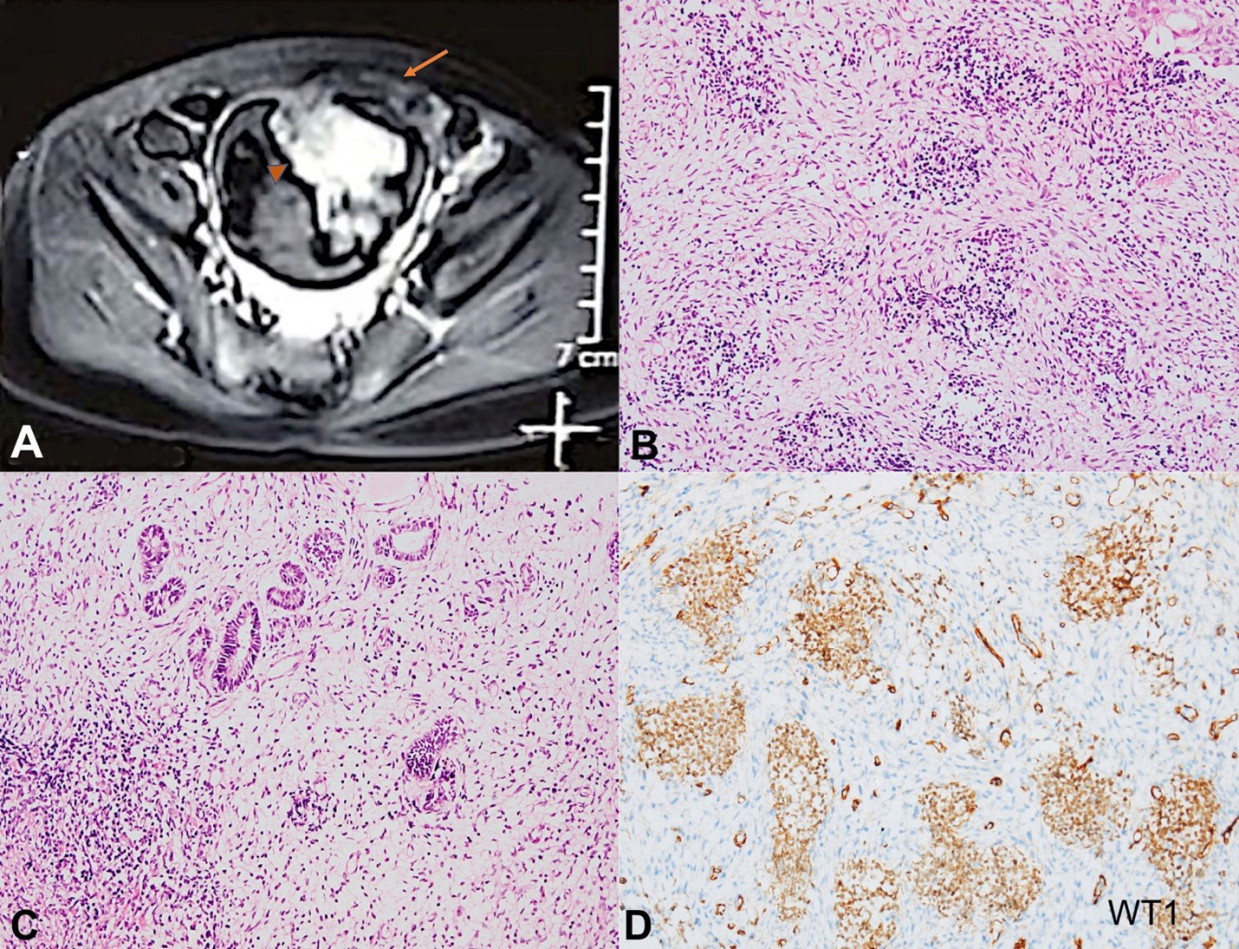
**A –** CEMRI with MR urography shows polypoidal, heterogeneous mass over the anterosuperior aspect of the urinary bladder wall. The mass with both endophytic (arrow head) and exophytic (arrow) growth is seen; B, C, and D are photomicrographs of the bladder tumor; **B and C** – Pretherapy cystoscopy guided biopsy showing triphasic component comprising blastemal (B; H&E, 200X), and epithelial and mesenchymal elements (**C**; H&E, 200X); **D** – The blastemal component positive for WT1 immunostain (200X).

A 4-year-old-female child presented with difficulty in micturition for one month along with straining, dribbling, and increased frequency of urination. There was intermittent gross hematuria and dysuria, however no history of abdominal distension or swelling elsewhere was noted. No history of fever, colicky lumbar pain, vomiting, oliguria, or peripheral edema was present. The abdominal examination lacked to reveal a palpable mass, distension, or tenderness. The baseline laboratory parameters were within normal limits. Ultrasonography (USG) of the abdomen showed a distended urinary bladder with a well-defined, oval-shaped, intraluminal mass measuring 3.4×1.7 cm. The contrast-enhanced Magnetic Resonance Imaging (CEMRI) with MR urography revealed a polypoidal, heterogeneous mass over the anterosuperior aspect of the urinary bladder wall (Figure 1A). The mass was both endophytic and exophytic measuring 5×4.9×5.2 cm and 2.1×2.3×2.1 cm, respectively. There was no significant pelvic, inguinal, or retroperitoneal lymph node enlargement. On imaging, no abnormality was noted in the kidneys, adrenal glands, liver, gallbladder, spleen, and pancreas. Cystoscopy-guided biopsies were sent for histopathological examination and the bladder was diagnosed with extrarenal WT. The patient received neoadjuvant chemotherapy [International Society of Pediatric Oncology for Wilms tumor (SIOP-WT-2001 protocol)] for 6 weeks. A six-week chemotherapy was administered against the usual 4 weeks owing to the unusual site of the tumor to make the tumor amenable to excision with minimal morbidity to the urinary bladder. A modest decrease in tumor size in response to the initial 4 weeks of chemotherapy with vincristine and actinomycin-D was noted. One dose of doxorubicin was administered at week 5. The computed tomography (CT) scan performed after 6 weeks of neo-adjuvant chemotherapy revealed a residual tumor of 2.5×2.0×1.8 cm and 2.0×2.5×2.7 cm, as the endophytic and exophytic components, respectively. The patient underwent laparoscopic tumor excision with bladder repair. The surgical specimen had all margins free of tumor, and histological risk was intermediate (mixed type). There were no significant lymph nodes along the bilateral iliac chain which could be sampled, consistent with the preoperative staging. As the staging of ERWT is not described, she was treated post-operatively with three drugs (vincristine, actinomycin-D, and doxorubicin) for 27 weeks without radiotherapy. She completed therapy in March 2021.

The initial biopsy on light microscopy revealed the fragmented bits showing focal preserved urothelial lining epithelium. The triphasic tumor contained undifferentiated blastema, primitive tubules, and admixed stroma was present in the subepithelial location. The blastemal component was placed randomly in a nesting pattern and was composed of primitive small round-to-oval blue cells with hyperchromatic nuclei, inconspicuous nucleoli, and scanty cytoplasm (Figure 1B). The small tubules were lined by cuboidal to columnar cells with hyperchromatic nuclei (Figure 1C). No primitive glomeruli were appreciated in this limited biopsy material. The stroma was edematous and comprised of a few discrete smooth muscle fibers. No definite foci of anaplasia were seen. No native detrusor muscle was included. WT1 immunostain highlighted the blastemal and epithelial components (Figure 1D).

Taken together with the clinical findings, radiological details and histo-morphological features, the diagnosis of extrarenal triphasic WT was offered.

After a few days, the child spontaneously expulsed a polypoidal tissue at home, which was passed out while urinating. This mass with compromised fixation measured 2.5×1.5×0.5 cm. Microscopically, the tumor fragments showed autolysis; however, in addition to the findings noted in the initial biopsy, occasional foci of cartilaginous islands were present. WT1 immunostain depicted the immunoreactivity in blastemal and epithelial components while the Myogenin, desmin, and Myo D1 were negative.

**Figure 2:**
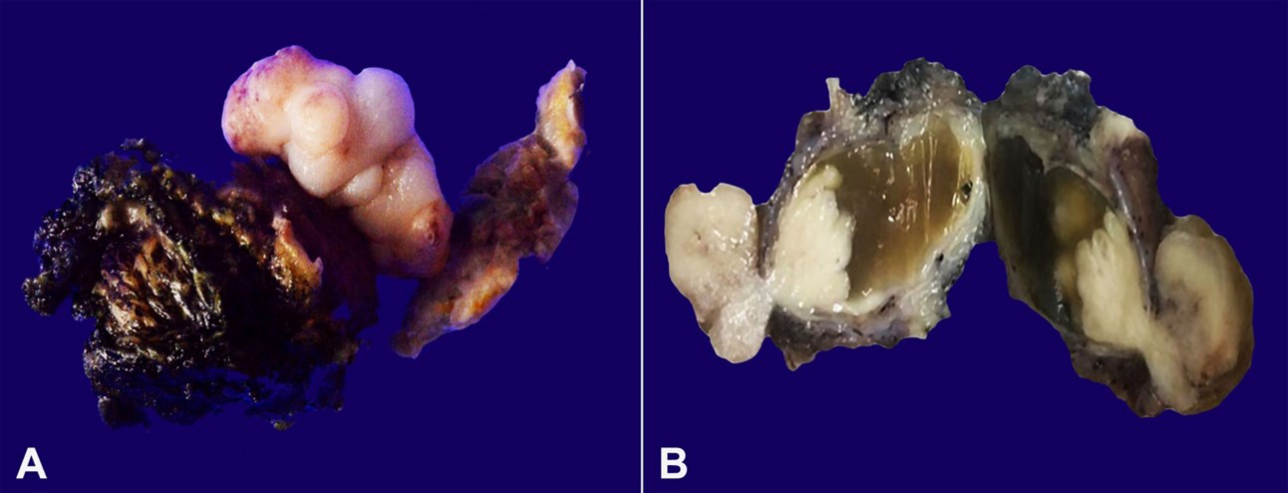
**A –** The partial cystectomy specimen reveals exophytic polypoidal growth; **B** – The cut surface shows both endophytic and exophytic growth with grey white and firm in appearance. The endophytic component is surrounded by the cystic space containing gel-like material.

Following chemotherapy, the partial cystectomy showed an exophytic, polypoidal mass (Figure 2A). The mass was seen arising from the mucosal aspect and measured 3.5×3×2 cm. On serial slicing, the cut surface also depicted the endophytic component measuring 1.5×1.5×1 cm protruding into the cystic space formed around it (Figure 2B).

**Figure 3:**
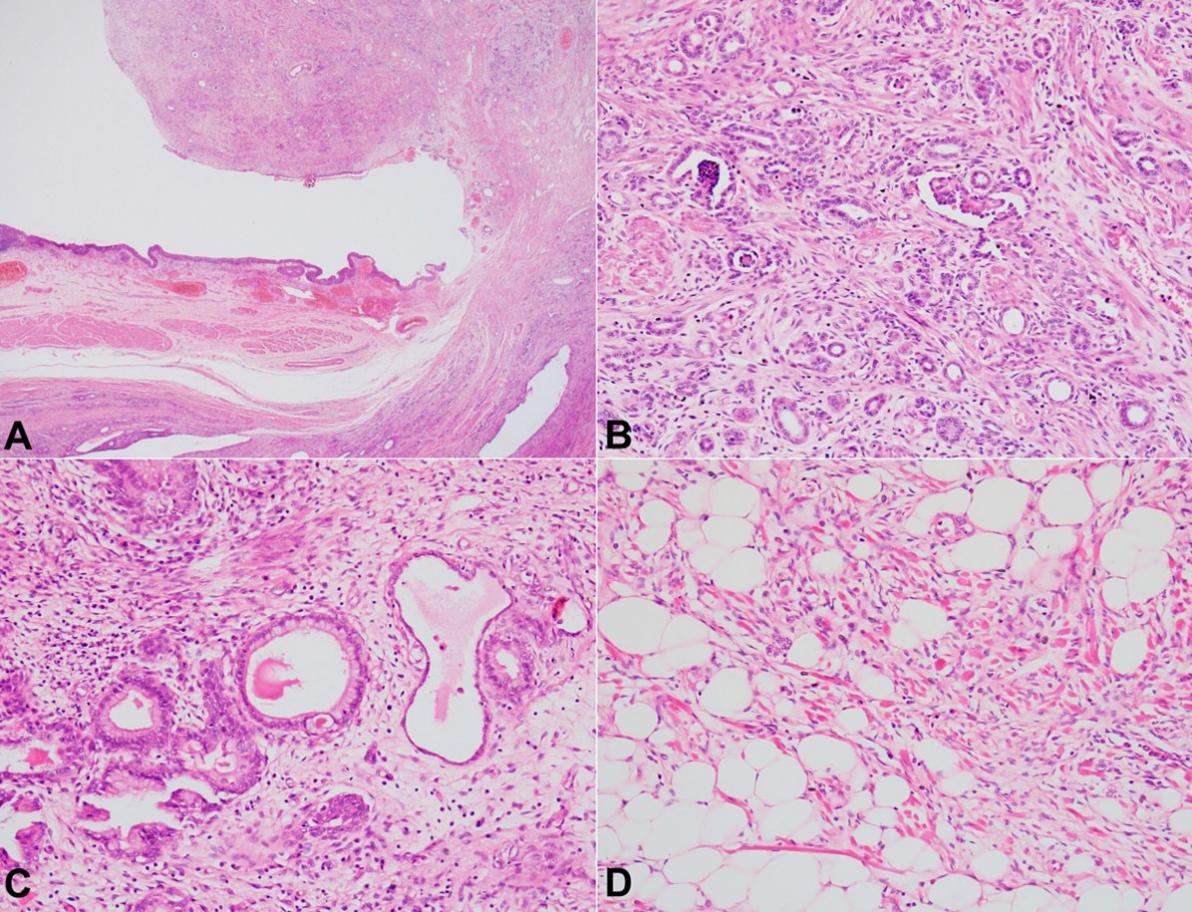
Photomicrographs of the partial cystectomy specimen showing: **A** – a polypoidal mass arising within the lumen. Bladder wall with detrusor muscle can be appreciated (right) (H&E, 40X); **B** – Biphasic component comprising of glomeruli, tubules, and smooth muscle fibers (H&E, 200X); **C** – Heterologous mucinous epithelium (H&E, 200X); **D** – Adipocytic and skeletal muscle differentiation (H&E, 200X).

Circumferentially, the tumor was 0.7 to 0.9 cm away from the surgical resection margins. Microscopically, the polypoidal mass was well-demarcated from the surrounding native bladder parenchyma by the fibrous pseudocapsule (Figure 3A). It was composed of an admixture of epithelial and mesenchymal elements. The epithelial component is comprised of primitive glomerular and tubular structures (Figure 3B). The tubules were small to cystic, lined by cuboidal to columnar cells, and depicted fine nuclear chromatin. Occasionally, they showed squamous and mucinous differentiation (Figure 3C). The stroma contained smooth muscle, fibroblasts, skeletal muscle, adipose tissue, ganglion cells, and occasional lymphoid aggregates (Figure 3D) with areas of myxoid and edematous change.

No definite foci of anaplasia or necrosis were noted. No blastemal element was identified even after thorough sampling and on WT1 immunostain. The absence of blastemal component and maturation of tumor components in the posttherapy surgical specimen indicated a tumor response to chemotherapy. The adjacent bladder wall showed foci of cystitis cystica and cystitis glandularis with prominent congested vascular channels in the lamina propria. No nephrogenic rests were seen. Thus, the tissue was diagnosed as primary bladder WT with favorable histology and free surgical margins.

She received adjuvant chemotherapy with a 3-drug regime (vincristine, actinomycin D, and doxorubicin) for 27 weeks without radiotherapy. She is doing well at 9 months post-completion of chemotherapy.

## DISCUSSION

Wilms tumor is one of the most common childhood malignancies, accounting for almost 95% of renal malignancies in Pediatrics. In 1961, Moyson et al. 6 described the extrarenal Wilms’ tumor (ERWT). The incidence of ERWT is estimated to range between 0.5 to 1% of all WT cases.^6^ The exclusion of intrarenal tumors and supernumerary kidneys is a necessity for imaging before diagnosing ERWT. The isolated urinary bladder as the primary site for ERWT is rare, and we witnessed three documented cases in the English literature on a thorough search in the PubMed database.

The age of presentation for ERWT is mostly childhood; however, few cases in adults have also been reported.^7^ The previously reported primary urinary bladder WT cases were seen in three male children (8 months,1 year, and 6 years old).^3^^-^ 5 The index patient presented at the age of 4 years. The clinical presentation of ERWTs usually depends on the location and stage of the tumor. The index case presented with bladder symptoms like the other reported cases.^3^^-^ 5 As the symptoms are not in concordance with classical WT a clinical diagnosis cannot be made, and the diagnosis of ERWT is achieved on histopathological examination.

The pathogenesis of ERWT remains indefinable, and several theories have been postulated. WT developing in ectopic nephrogenic rest has been considered the most common and acknowledged hypothesis.^8^ The pathogenesis in the reported cases was unclear, and both the authors as well as we kept the possibility of originating from residual immature nephrogenic cells: pluripotent embryonic renal precursor cells or metanephric blastemal.^3^^,^ 4 To diagnose ERWT as primary, Beckwith and Palmer^9^ proposed the pathological criteria that include the documentation of classic triphasic WT pattern outside the kidneys in the absence of teratoid or anaplastic elements, and both kidneys are tumor free on imaging (multislice spiral CT).

It was recently concluded that the teratoid WT should be considered an extreme spectrum of WT rather than a distinct entity as they do not carry any prognostic significance.^10^ There are no consistent universal criteria in the literature; however, the presence of heterologous elements (HE) in >50% of the tumor or more than or equal to three heterologous elements within the tumor may help spotting it.^10^^,^ 11 We came across a case of WT with heterologous differentiation that has not been reported in the previous case reports of primary urinary bladder WT.^3^^-^ 5 Differentiating nephrogenic rests (NR) from the ERWT is not an easy task as the described histological criteria in the literature are not conclusive, especially in a post-chemotherapy specimen. Even no definite molecular studies are available. The features like usual solitary nature, fibrous capsule, anaplasia, and skeletal muscle differentiation may favor WT over NR. The possibility of NR in our patient was not considered as the presence of most of the above-mentioned findings favored WT. Similarly, NR was not identified even in the reported cases of primary bladder WTs.^3^^-^ 5

There are no accepted staging or treatment guidelines for ERWT. However, as per the National Wilms Tumor Study (NWTS), all ERWTs are considered Stage II or higher.^12^^,^ 13 The reported cases and our patient, received a chemotherapy regime. They underwent the tumor surgical excision, except the case reported by Ismy et al.^4^ On follow-up, both the documented cases, including the index patient, were doing well.

In conclusion, primary bladder WT is an extremely uncommon entity. It must be considered in the differential of a small blue round cell tumor at the extrarenal site in the pediatric age group. The diagnosis is especially challenging in small biopsy material, although it has immense significance in management and prognosis. An array of immunohistochemical stains along with clinico-pathological correlation and awareness about the entity help in the diagnosis of this uncommon tumor.

## References

[1] Webber BL, Parham DM, Drake LG, Wilimas JA (1992). Renal tumors in childhood. Pathol Annu.

[2] Goldblum JR, Lamps LW, McKenney JK, Myers JL Rosai and Ackerman’s Surgical Pathology.

[3] Zhang DY, Lin T, Wei GH (2010). A rare case of simultaneous occurrence of Wilms’ tumor in the left kidney and the bladder. Pediatr Surg Int.

[4] Ismy J, Ismy J, Kamarlis R, Mustafa A (2019). Rare case of primary bladder Wilm’s tumor in a 1-year-old boy. Urol Case Rep.

[5] Sindhu II, Saeed H, Wali R, Mehreen A (2019). Primary extra-renal Wilms’ tumor in urinary bladder: rare presentation of a common pediatric malignancy. J Coll Physicians Surg Pak.

[6] Moyson F, Maurus-Desmarez R, Gompel C (1961). Mediastinal Wilms’ tumor?. Acta Chir Belg.

[7] Armanda V, Culić S, Pogorelić Z, Kuljiš D, Budimir D, Kuzmić-Prusac I (2012). Rare localization of extrarenal nephroblastoma in 1-month-old female infant. J Pediatr Urol.

[8] Wu Y, Zhu X, Wang X, Wang H, Cao X, Wang J (2014). Extrarenal nephroblastomatosis in children: a report of two cases. BMC Pediatr.

[9] Beckwith JB, Palmer NF (1978). Histopathology and prognosis of Wilms tumors: results from the First National Wilms’ Tumor Study. Cancer.

[10] D’Hooghe E, Mifsud W, Vujanić GM (2019). “Teratoid” Wilms Tumor: The extreme end of heterologous element differentiation, not a separate entity. Am J Surg Pathol.

[11] Fernandes ET, Parham DM, Ribeiro RC, Douglass EC, Mahesh Kumar AP, Wilimas J (1988). Teratoid Wilms’ tumor: the St Jude experience. J Pediatr Surg.

[12] Shojaeian R, Hiradfar M, Sharifabad PS, Zabolinejad N, van den Heuvel-Eibrink MM (2016). Wilms tumor.

[13] Madanat F, Osborne B, Cangir A, Sutow WW (1978). Extrarenal Wilms tumor. J Pediatr.

